# Supplementary material for: Inverse Correlation of Th2-Specific Cytokines with Hepatic Egg Burden in S. mansoni-Infected Hamsters
Source: Cells. 2024 Sep 20;13(18):1579. doi: 10.3390/cells13181579 (PMC11430739; doi:10.3390/cells13181579)
Supplement: Supplementary file 1 [file cells-13-01579-s001.zip › cells-3209420-supplementary.pdf]

cells
MDPI

Supplementary Material

Inverse Correlation of Th2-Specific Cytokines with Hepatic Egg Burden in *S. mansoni*-Infected Hamsters

Lena Russ [1,†], Verena von Bülow [1,†], Sarah Wrobel [1], Frederik Stettler [1], Gabriele Schramm [2], Franco H. Falcone [3], Christoph G. Grevelding [3], Martin Roderfeld [1] and Elke Roeb [1,*]

[1] Department of Gastroenterology, Justus Liebig University, 35392 Giessen, Germany; lena.russ@gmx.de (L.R.); verena.von-buelow@innere.med.uni-giessen.de (V.v.B.); martin.roderfeld@innere.med.uni-giessen.de (M.R.)
[2] Early Life Origins of Chronic Lung Diseases, Priority Research Area Chronic Lung Diseases, Research Center Borstel, Leibniz Lung Center, 23845 Borstel, Germany
[3] Institute of Parasitology, BFS, Justus Liebig University, 35392 Giessen, Germany; franco.falcone@vetmed.uni-giessen.de (F.H.F.); christoph.grevelding@vetmed.uni-giessen.de (C.G.G.)
* Correspondence: elke.roeb@innere.med.uni-giessen.de
† These authors contributed equally to this work.

Content:

Supplementary Figure S1: Varying degrees of egg load correlated with worm burden in hamster liver and colon.
Supplementary Table S1: Primer list.

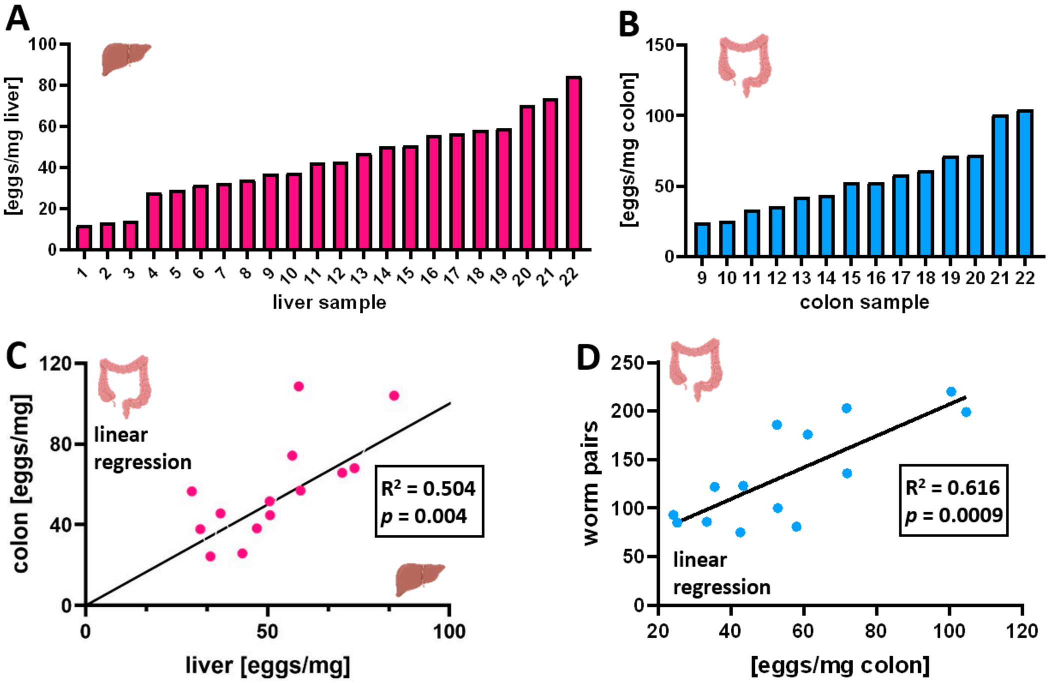

Supplementary Figure S1: Varying degrees of egg load correlated with worm burden in hamster liver and colon. (A and B) Spectrum of egg load in the liver and colon of *S. mansoni*-infected hamsters. (C) The individual numbers of eggs in liver and colon correlated well. (D) Linear correlation of egg burden in colon tissue and the number of worms that were isolated by perfusion.

Supplementary Table S1: Primer list

Il4 sense: 5'-ggg tct cag gcc cca gct ag-3', antisense: 5'-ttg cga agc acc tgg gaa gc-3'

Il5 sense: 5'-aac gag acg gtg agg ctt cct-3', antisense: 5'-cgc ctc tcc tgg cca cac tg-3'

Il10 sense: 5'-tcc gag agc tga gga ctg cc-3', antisense: 5'-tgg ttc tct gcc tgg ggc at-3´

Il13 sense: 5'-aat ggc ggg ttc tgt gca gc-3', antisense: 5'-tga tgc cct tcg gac gca ga-3'

Tnfα sense: 5'-cac tca cac tca gat cat ctt ct-3', antisense: 5'-gct aca acg tgg gcc aca g-3'

Ifnγ sense: 5'-cca ggc cat cca gag gag ca-3', antisense: 5'-cca ccc cca aaa cag cac cg-3'

Ipse/α1 sense: 5'-gcg ttg gct cac tct cac cac c-3', antisense: 5'-aca gta tgt cct tct ccg ttt cgg t-3'

Omega1 sense: 5'-gga cgg aga ggg atg tat ca-3', antisense: 5'-ttc caa gga acg ggc agt-3'

Kappa5 sense: 5'-acg ctg cta gat caa ctt gg-3', antisense: 5'-tgg ttt tgt tca cac cca ca-3'
